# Identification and Expression Analysis of Sulfate Transporter Genes Family and Function Analysis of *GmSULTR3;1a* from Soybean

**DOI:** 10.3390/ijms25169080

**Published:** 2024-08-21

**Authors:** Jingwen Zhou, Yue Dong, Yue Liu, Yifan Huang, Wenjing Jiang, Xiangmin Zheng, Huimin Zhang, Na Gong, Xi Bai

**Affiliations:** College of Life Science, Northeast Agricultural University, Harbin 150001, China; 18846084378@163.com (J.Z.); dydeyx321@163.com (Y.D.); 18646370430@163.com (Y.L.); 19819512772@163.com (Y.H.); wj01250414@163.com (W.J.); zxm1487611887@outlook.com (X.Z.); zhanghuimin202202@163.com (H.Z.); 15342825207@163.com (N.G.)

**Keywords:** sulfate transporter genes, sulfur nutrition, soybean, sulfur-containing amino acids, abiotic stress

## Abstract

Sulfate transporters (SULTRs) are essential for the transport and absorption of sulfate in plants and serve as critical transport proteins within the sulfur metabolism pathway, significantly influencing plant growth, development, and stress adaptation. A bioinformatics analysis of SULTR genes in soybean was performed, resulting in the identification and classification of twenty-eight putative GmSULTRs into four distinct groups. In this study, the characteristics of the 28 *GmSULTR* genes, including those involved in collinearity, gene structure, protein motifs, *cis*-elements, tissue expression patterns, and the response to abiotic stress and plant hormone treatments, were systematically analyzed. This study focused on conducting a preliminary functional analysis of the *GmSULTR3;1a* gene, wherein a high expression level of *GmSULTR3;1a* in the roots, stems, and leaves was induced by a sulfur deficiency and *GmSULTR3;1a* improved the salt tolerance. A further functional characterization revealed that *GmSULTR3;1a-*overexpressing soybean hairy roots had higher SO_4_^2−^, GSH, and methionine (Met) contents compared with the wild-type (WT) plant. These results demonstrate that the overexpression of *GmSULTR3;1a* may promote the sulfur assimilation metabolism and increase the content of sulfur-containing amino acids in plants.

## 1. Introduction

Sulfur plays a crucial role in plant growth, development, and stress responses, serving as an integral component of various plant compounds and being indispensable for numerous physiological processes [1]. Sulfur helps plants manage stress and is crucial for producing glutathione, which maintains cell balance and reduces oxidative stress [2,3,4]. Sulfate is the anionic form of sulfur that affects plant responses to ABA (abscisic acid)-related drought and salinity [5,6].

Plants absorb sulfate from the soil through sulfate transporters (SULTRs) [7]. The SULTR gene family has 12, 8, 12, 12, 11, 16, 22, 15, and 9 members in *Arabidopsis*, maize, rice, potato, sorghum, *Populus stremula* × *P. alba*, wheat, and apple, respectively [8,9,10,11], while the *Camelina sativa* and *Brassica napus* genomes contain 36 and 45 putative SULTR genes, respectively [12], and tea and cotton plants have 8 and 106 SULTR genes, respectively [13,14]. All the identified SULTRs have 12 transmembrane domains and one STAS domain at the end [15]. The SULTR family in Arabidopsis, which includes the high-affinity transporters *AtSULTR1;1* and *AtSULTR1;2*, efficiently uptakes sulfate into the roots from the soil [16]. Source-to-sink sulfate transport is facilitated by *AtSULTR1;3*, which is found in the phloem [17]. Two low-affinity members of the SULTR2 subfamily, *AtSULTR2;1* and *AtSULTR2;2*, play a crucial role in the transportation of sulfate to the xylem [18]. The members of the SULTR3 subfamily are functionally diversified, such that *AtSULTR3;1*, *AtSULTR3;2 and AtSULTR3;3* tend to be expressed in the leaves [19], and *AtSULTR3;5* enhances sulfate transport from the roots to the shoots via *AtSULTR2;1* [20]. *AtSULTR3;2*, *AtSULTR3;3*, *AtSULTR3;4*, and *AtSULTR3;5* supply sulfate to maturing embryos [21], while *AtSULTR4;1* and *AtSULTR4;2* facilitate sulfate efflux from vesicles [22]. In another study, maize was shown to have eight putative SULTR genes induced by drought and heat stress, except for *ZmSULTR3.3* [15]. Potatoes have twelve SULTR genes, with the *StSULTR3s* potentially involved in drought and salt stress responses [8]. Wheat has 22 *TdSULTR* genes, with their expression induced under S starvation [23]. A particular class of putative SULTR genes that does not seem to transport sulfate, *OsSULTR3;3*, is involved in the transportation of phosphorus (P) to the phytic acid synthesis pathway [24,25], and *OsSULTR3;4* controls the allocation of phosphorus to the grain [26]. Soybeans are a key source of oil and protein, but their low levels of sulfur-containing amino acids can cause nutritional imbalances; studies have shown that *GmSULTR1;2b* in soybean helps with sulfur absorption and plant tolerance to sulfur deficiency stress [16].

Our comprehension of the characterization of soybean sulfate transporters remains constrained. This study offers an in-depth investigation of the soybean SULTR family, encompassing both their characteristics and expression profiles. Furthermore, functional studies were conducted on *GmSULTR3;1a* in soybean to investigate its role in sulfate transport and the enhancement of the soybean methionine content. These findings lay the groundwork for future investigations into the function of soybean SULTR genes.

## 2. Results

### 2.1. Identification of the GmSULTR Gene Family and Syntenic Analysis of SULTR Genes in Soybean

A total of 12 *Arabidopsis* SULTR proteins were used to search for putative *GmSULTR* genes in *Glycine max*. In total, 28 *GmSULTR* genes were identified; these were also reported in Ding’s study [16]. The 28 putative soybean SULTR proteins were classified into four groups, consistent with studies on other species [1,8,9,10,11]. The subcellular localization predictions showed that *GmSULTR4;2* and *GmSULTR2.1c* were predicted to localize to the chloroplast, while the other 26 *GmSULTRs* had cellular membrane localization (Table S1).

**Figure 1:**
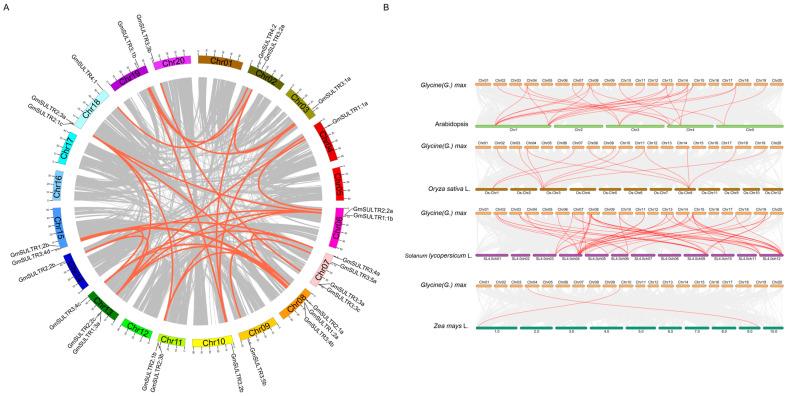
Chromosome location, collinearity and synteny analysis of *GmSULTR* genes. (**A**) Chromosomal location of the *GmSULTR* gene family in Glycine (G.) max. and collinearity analysis of *GmSULTRs*. Gray lines represent all collinear blocks in the genome of *Glycine max*; red lines represent duplicated *GmSULTR* gene pairs. (**B**) Synteny analysis of *GmSULTR* genes between *Glycine max* and four other species. The gray lines indicate the gene blocks in *Glycine max* that are orthologous to the other genomes. The red lines delineate syntenic *SULTR* gene pairs.

Subsequently, to identify duplication events within the *GmSULTR* gene family, a collinearity analysis was conducted utilizing the MCScanX program within Tbtools-II v2.096. Our analysis of gene duplication events revealed that all *GmSULTR* genes are the result of segmental duplications (Figure 1A). The syntenic relationships between *GmSULTRs* and the *SULTR* genes from four other plant species (*Arabidopsis*, rice, wheat, and tomato) were analyzed, and the results showed that there were 26, 13, 2, and 50 orthologous gene pairs between soybean and these four species (*Arabidopsis*, rice, wheat, tomato), respectively (Figure 1B). This indicated that soybeans are more closely related to *Arabidopsis* and tomato than the other plants, which is consistent with the degree of relationship based on the sequence similarity.

A further analysis of these collinear genes revealed that some *GmSULTR* genes existed in more than one collinear gene pair in four species, such as *GmSULTR3;2b* and *GmSULTR1;1a*, and that 16 *GmSULTR* genes showed collinearity with *Arabidopsis* (16 genes, namely *GmSULTR1;1a*, *1;2b*, *2;1a*, *2;1b*, *2;2b*, *2;2c*, *2;3a*, *3;1a*, *3;1b*, *3;2a*, *3;2b*, *3;3c*,*3;4a*, *3;4b*, *3;4c*, *and 3;4d,* as shown in Table S2); in total, 22 *GmSULTR* genes showed collinearity with tomato (Table S2). The number of genes with collinearity to the dicots was much higher than that with collinearity to the monocots.

### 2.2. Analysis of Protein Conserved Motisf, Gene Structure, and Cis-Elements of SULTRs in Soybean

**Figure 2:**
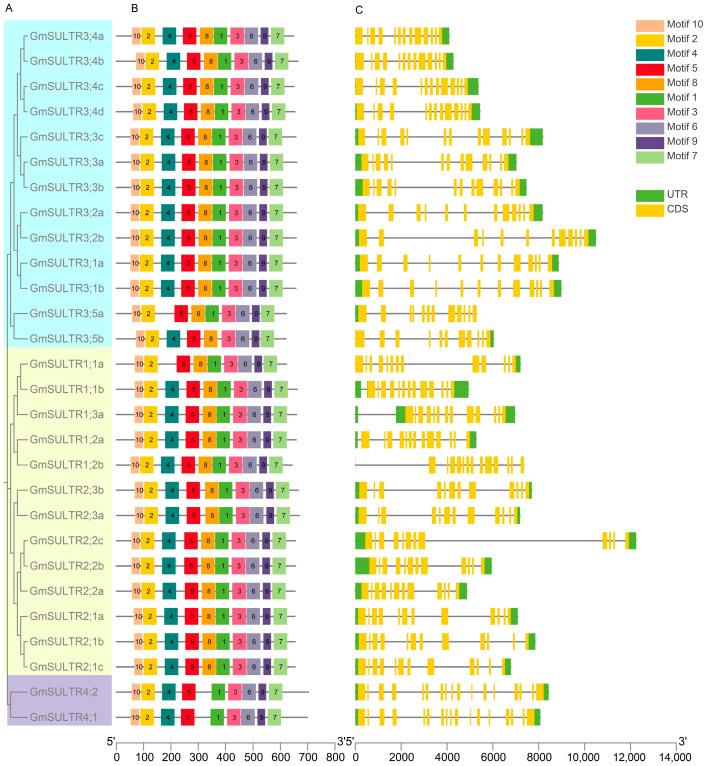
Conserved motif and gene structure analysis of *GmSULTRs* genes in *Glycine max*. (**A**) Phylogenetic tree of all GmSULTR proteins. (**B**) Motif distribution of GmSULTR proteins; motifs 1–10 are shown as rectangular boxes of different colors. (**C**) Gene structures of *GmSULTR* genes arranged according to the phylogenetic relationship; green boxes represent 5′UTR and 3′UTR, yellow boxes represent exons, and gray lines represent introns.

To identify conserved motifs within the GmSULTRs, the complete protein sequences were subjected to analysis using the MEME program. A total of 10 individual motifs were characterized. Furthermore, it was observed that the majority of GmSULTR family members shared similar conserved motifs (Figure 2B). All *GmSULTR* proteins contained motifs 2–3, 5–7 and 9–10; *GmSULTR1;1a* and *GmSULTR3;5a* lacked motif 4, *GmSULTR3;5b* lacked motif 1, and *GmSULTR4;1* and *GmSULTR4;2* lacked motif 8.

To elucidate the genomic patterns, the organization of exons and introns was analyzed. The number of exons ranged from 11 to 17. Notably, the *GmSULTRs* in group 2 were conserved, each containing 12 exons. In contrast, *GmSULTR4;1* and *GmSULTR4;2* in group 4 exhibited the highest exon count, with 17 exons. Interestingly, in contrast to the similar exon lengths of *GmSULTRs* within each cluster, the intron lengths varied significantly (Figure 2C). The gene structure of closely related members was more similar.

**Figure 3:**
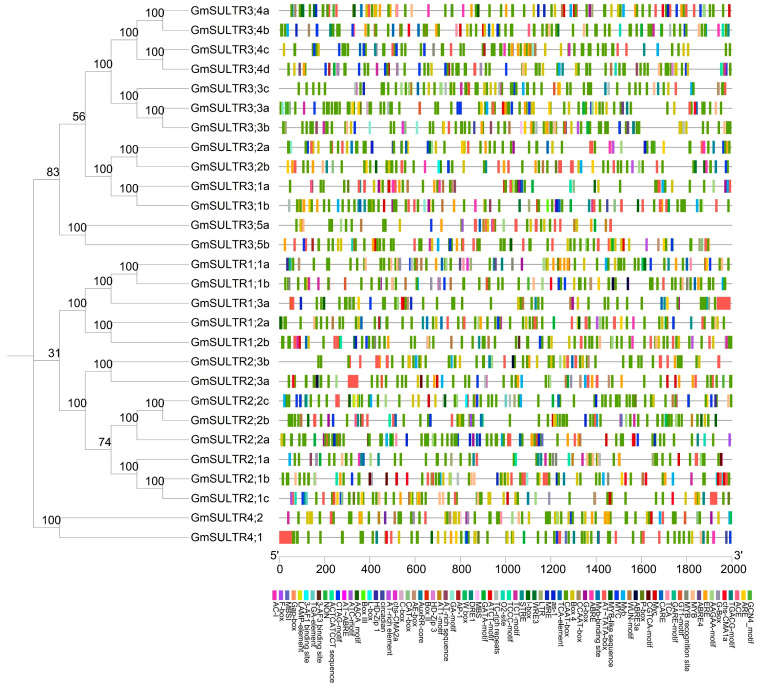
*Cis* elements in the promoters of *GmSULTR* genes. The black line indicates the length of the *GmSULTR* gene promoter. The rectangular boxes with different colors represent different types of *cis*-acting elements.

We conducted an analysis of the cis-regulatory elements within the 2000 bp promoter sequence of GmSULTR genes using the PlantCARE database to elucidate their transcriptional regulatory mechanisms and potential functions (Figure 3). Our focus was on elements associated with hormonal responses, stress, growth, and development. Specifically, ABRE, GARE, TGACG-motif, TCA-element, and ERE were identified in groups 1 through 4. Stress-related elements such as MBS, MYB, MYC, LTR, DRE, ARE, WUN-motif, and TC-rich repeats [27] were detected in groups 1 through 3, while MBS, MYC, ARE, and WUN-motif were identified in group 4. The growth and development-related elements, including the CAT box, MBSI, GCN4-motif, and circadian, were identified across various groups. Specifically, the GCN4-motif and circadian elements were detected in group 1, while the CAT box and MBSI elements were present in groups 3 and 4. Additionally, light-responsive elements, such as the Box 4 and G-Box, were observed in groups 1, 2, and 3. These findings suggest that the *GmSULTR* gene family may be involved in hormone metabolism and stress response.

### 2.3. Expression Profiling of GmSULTRs in Various Tissues

**Figure 4:**
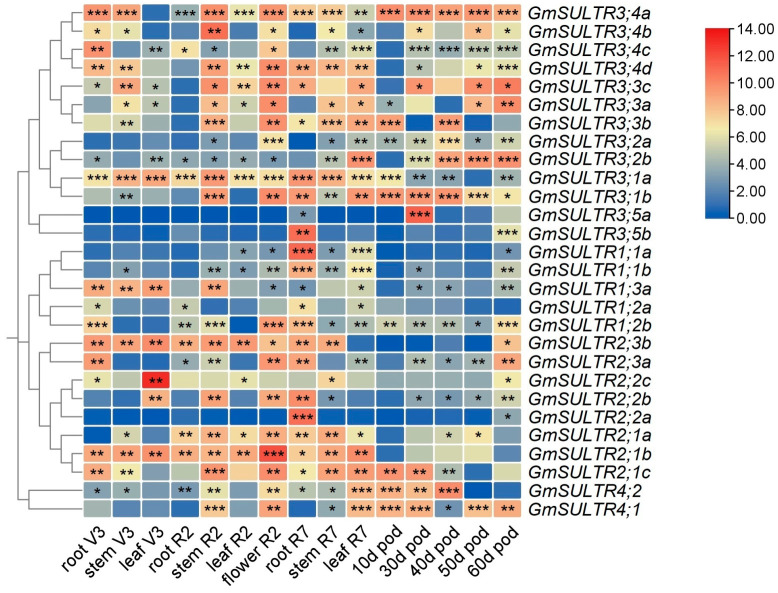
The qRT-PCR analysis of *GmSULTRs* in different tissues of *Glycine max*. The gene evolutionary relationships are on the left, the tissue names are at the bottom of the figure, and the expression abundance of each transcript is represented by the bar color: red, higher expression; blue, lower expression. Note: V3 (the third trifoliolate leaf expansion), R2 (the blooming period), and R7 (the initiation of maturity) are growth stages. The stars indicate a significant difference (* *p* < 0.05, ** *p* < 0.01, and *** *p* < 0.001) compared to the control.

Assessing gene expression patterns can be a valuable tool in predicting the potential biological functions of genes [28]. In this study, the potential function of *GmSULTRs* in soybean was investigated by analyzing the expression patterns of 28 *GmSULTRs* in various tissues, such as the roots, stems, leaves, and pods, using quantitative real-time polymerase chain reaction (qRT-PCR). Our findings indicated that all 28 GmSULTRs exhibited differential expressions across soybean tissues (Figure 4). In group 3, *GmSULTR3; 4a* demonstrated the highest expression levels in the pods (10d); *GmSULTR3;2b, GmSULTR3;3a,* and *GmSULTR3;3c* were most expressed in 60 d pods; *GmSULTR3;1a* showed high transcript levels in the roots, stems, and leaves; and the expression levels of *GmSULTR3; 2a and GmSULTR3;5b* were relatively low in the different tissues. In group 1, *GmSULTR1;1a* and *GmSULTR1;1b* were at relatively high levels in the root (R7), and *GmSULTR1;3a* exhibited relatively high expression levels in the roots, stems and leaves of V3. In group 2, *GmSULTR2;1b* and *GmSULTR2;2c* had high expression levels in the flowers (R2) and leaves (V3), respectively. Additionally, *GmSULTR4;1* and *GmSULTR4;2* in the group 4 family demonstrated elevated expression levels in the pods. Based on the different expression patterns of *GmSULTRs*, it is possible that *GmSULTRs* may play different roles during growth and development.

### 2.4. GmSULTRs Expression in Response to Abiotic Stress and Plant Hormones Treatments

**Figure 5:**
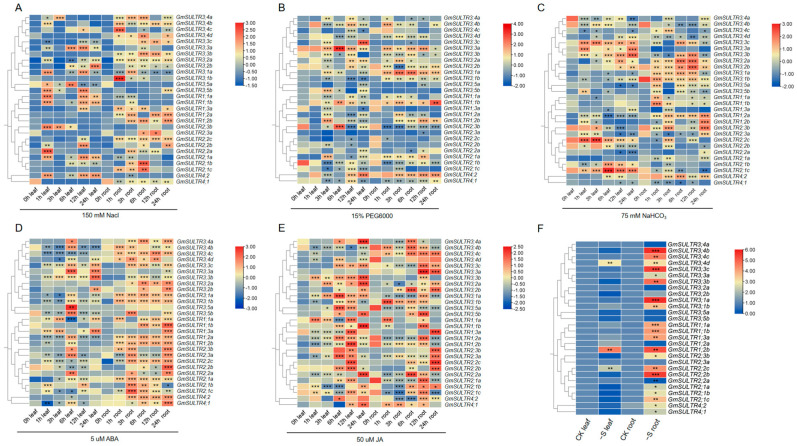
Expression patterns of *GmSULTR* genes following exogenous treatments. The expression levels of the *GmSULTR* genes under 200 mM NaCl (**A**),15% PEG6000, (**B**), 75 mM NaHCO_3_ (**C**), 5 µM ABA (**D**), 50 µM MeJA (**E**), and sulfur deficiency (**F**) treatments using a qRT-PCR analysis. The heatmap was constructed based on the expression level of each gene in the leaves and roots relative to that of *GmTUA5*. The blue and red boxes indicate lower and higher expression levels, respectively. The scale bar represents the fold change (log_2_ value). Stars indicate a significant difference (* *p* < 0.05, ** *p* < 0.01 and *** *p* < 0.001) compared to the control.

To investigate the potential roles of *GmSULTRs* in response to abiotic stress and various plant hormones, soybean seedlings (V3) were exposed to high-salinity (200 mM NaCl), drought (15% PEG6000) or alkali conditions (75 mM NaHCO_3_), or ABA (5 µM) or MeJA (50 µM). As illustrated in Figure 5A, *GmSULTR3;1a*, *GmSULTR3.5b*, *GmSULTR1.1a*, *and GmSULTR1.1b* were induced markedly in the 1 h and 12 h leaves in response to high salinity. In contrast, only the expressions of *GmSULTR3;1b*, *GmSULTR3;4b*, and *GmSULTR3;4c* showed a highly significant increase in the 1 h roots. *GmSULTR1.2a*, *GmSULTR1.2b*, *and GmSULTR3;1a* were highly expressed in the 3 h roots.

Drought stress up-regulated *GmSULTR3;3a* in the leaves and roots, *GmSULTR3;3c* in the leaves, and *GmSULTR3;1a* in the roots. The genes that were down-regulated by the drought treatment included *GmSULTR1;3a*, *GmSULTR2;1b*, *GmSULTR2;2b*, and *GmSULTR2;3c* (Figure 5B). When the seedlings were exposed to alkali stress, the expression of *GmSULTR2;1c* and *GmSULTR3;3a-3;3c* in the leaves and *GmSULTR1;1a*, *GmSULTR1;1b*, *GmSULTR1;2a*, *GmSULTR3;1a*, *and GmSULTR3;2a* in the roots markedly increased, while that of *GmSULTR2;1c*, *GmSULTR3;3a*, *GmSULTR1;3a*, and *GmSULTR4;1* in the roots was inhibited (Figure 5C).

To study how *GmSULTR* genes respond to changes in sulfur deficiency, qPCR was used to analyze their transcript levels in the roots and leaves during the V3 stage under sulfur deficiency. As depicted in Figure 5F, under sulfur-deficient conditions, the expression of *GmSULTR1;2b*, *GmSULTR2;2b*, *GmSULTR3;1a*, *GmSULTR3;3c*, and *GmSULTR3;4b* exhibited a high transcript abundance in the roots. These results imply that these *GmSULTRs* might participate in the responses to sulfate deprivation.

The expression of certain genes can be modulated by hormonal signals. As for the ABA treatment, most *GmSULTR* genes showed a visible increase in the roots (Figure 5D). The expressions of *GmSULTR2;2a*, *GmSULTR3;3a*, *GmSULTR3;5a*, and *GmSULTR3;5b* were increased significantly in the 6 h leaves. Under the MeJA treatment, *GmSULTR1;1b*, *GmSULTR3;2a*, *GmSULTR3;3b*, *GmSULTR3;4a*, and *GmSULTR3;5b* were induced in the 24 h leaves, while *GmSULTR1;1a* and *GmSULTR2;2a* were inhibited in the 6h leaf. However, *GmSULTR1;1a*, *GmSULTR1;1b*, *GmSULTR3;1a*, *GmSULTR3;3a*, *GmSULTR3;3c*, and *GmSULTR3;5b* showed a substantial increase in the 12 h roots, while *GmSULTR1;3a*, *GmSULTR2;2c*, *GmSULTR2;3a*, *and GmSULTR2;3b* exhibited a significant increase in expression in the 24 h roots. Meanwhile, the expression of *GmSULTR2;1c* and *GmSULTR3;2a* declined in the 3 h roots (Figure 5E). Overall, most *GmSULTRs* proved to be sensitive to all five treatments and were found to potentially play a major role under conditions of abiotic stress.

### 2.5. GmSULTR3;1a Improved Salt Tolerance

**Figure 6:**
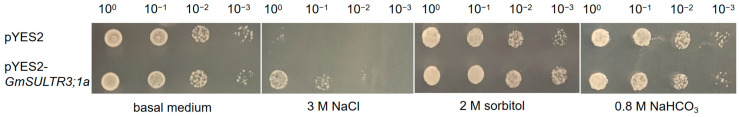
Salt tolerance test of yeast cells expressing *GmSULTR3;1a*. The effects of the ectopic overexpression of the full length of *GmSULTR3;1a* were examined. Serially diluted (10× fold) cells were spotted and the growth of the spotted cells was examined after incubation on basal medium and after being supplied with basal medium, with 3 M Nacl, 2 M sorbitol and 0.8 M NaHCO_3_.

In the realm of investigating the sulfur transporter proteins within soybean, the focus on the SULTR3 group has been comparatively scarce. The SULTR3 family constitutes the most extensive group of sulfur transporter proteins in soybeans. In this research, *GmSULTR3;1a* exhibited a notably elevated expression across the entire developmental cycle, particularly in the roots, stems, and leaves (Figure 4). Furthermore, the expression of this gene in both the roots and leaves was observed to be triggered and enhanced in response to various stress treatments (Figure 5). To elucidate the potential function of *GmSULTR3;1a* in response to abiotic stress tolerance, we analyzed yeast cells with the ectopic expression of *GmSULTR3;1a* in a basal medium with 3 M Nacl, 0.8 M NaHCO_3_ and 2 M sorbitol. Yeast cells transformed with an empty pYES2 vector were used as the control. The ectopic expression of the *GmSULTR3;1a* gene enhanced the salt tolerance of yeast cells compared with the control yeast cells (Figure 6). We developed *GmSULTR3;1a*-overexpressed soybean hairy root lines (comprising lines 3, 4, and 12) (Figure S1) and subjected them to a 150 mM salt treatment. Our observations indicated that the degree of wilting in wild-type soybean was significantly more severe than that in the hairy root lines overexpressing *GmSULTR3;1a*. This finding further substantiates the enhanced salt resistance conferred by the *GmSULTR3;1a* transgene (Figure S2).

### 2.6. GmSULTR3;1a Alters Sulfur-Containing Compounds and Root Phenotypes of Transgenic Soybean Hairy Roots

**Figure 7:**
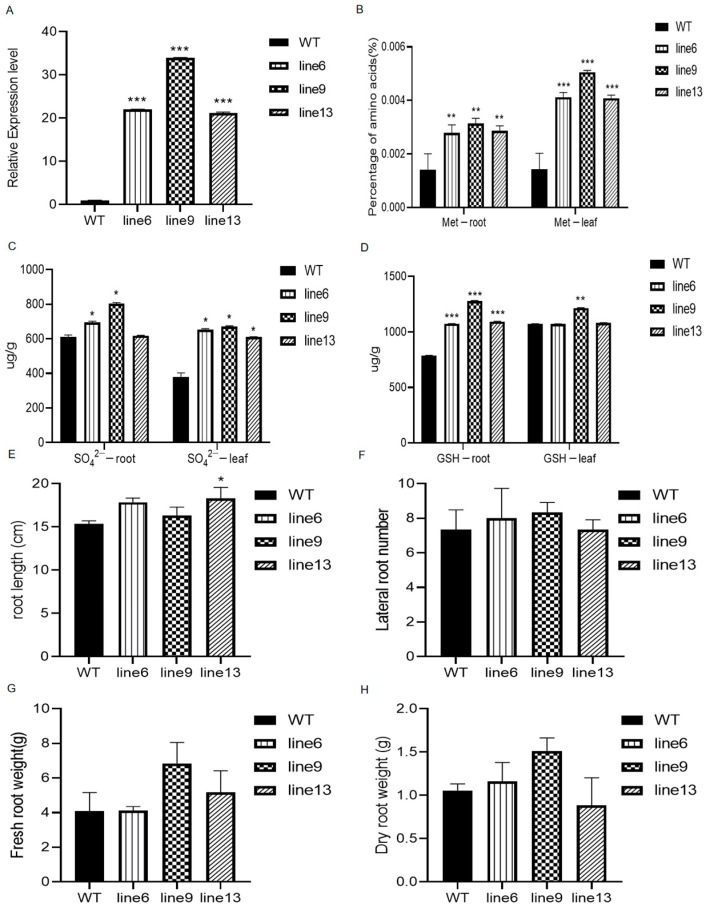
Sulfur-containing compounds and root phenotypes of *GmSULTR3;1a-*transgenic soybean hairy roots. (**A**) qRT-PCR analysis of *GmSULTR3;1a* transcript levels in transgenic hairy roots. Methionine content (**B**), sulfate ion content (**C**), and GSH content (**D**) of roots and leaves in *GmSULTR3;1a-*transgenic soybean hairy root material. Root length (**E**), lateral root number (**F**), fresh weights (**G**), and dry weights (**H**) of roots in *GmSULTR3;1a-* transgenic soybean hairy roots. Stars indicate a significant difference (* *p* < 0.05, ** *p* < 0.01 and *** *p* < 0.001) compared to the control.

To investigate the function of *GmSULTR3;1a* in the synthesis of sulfur-containing amino acids, we constructed *GmSULTR3;1a* over-expressing transgenic soybean hairy root lines. The successful transformation of *GmSULTR3;1a* into soybean hairy roots was verified using qRT-PCR. The qRT-PCR assays demonstrated that *GmSULTR3;1a* was transcribed into transgenic soybean hairy root line 6 (21-fold), line 9 (34-fold) and line 13 (21-fold) (Figure 7A). The contents of methionine (Figure 7B), SO_4_^2−^ (Figure 7C), and GSH (Figure 7D) were significantly higher than those in the WT plant. Particularly in line 9, the methionine content was observed to be 2.2-fold higher in the roots and 3.5-fold higher in the leaves compared to the WT plant. Similarly, the SO_4_^2−^ in line 9 was elevated by 1.3-fold in the roots and 1.8-fold in the leaves. Additionally, the GSH content exhibited an increase of 1.2-fold in the roots and 1.1-fold in the leaves relative to the WT plant. There was no significant increase in the root length, lateral root number, fresh weights, or dry weights in the *GmSULTR3;1a-*transgenic soybean hairy roots compared with the wild type (Figure 7E–H). In summary, overexpressing *GmSULTR3;1*a led to higher SO_4_^2−^ and methionine levels by enhancing SO_4_^2−^ absorption, boosting sulfur metabolism, and increasing the amino acid content in plants.

## 3. Discussion

Sulfur is an indispensable nutrient that significantly contributes to various vital growth processes and metabolic functions in plants [29]. Without an adequate supply of sulfur, plants may exhibit stunted growth, the yellowing of leaves, and a decreased resistance to environmental pressures. Therefore, ensuring sufficient sulfur levels is vital for promoting optimal plant health and productivity [30]. This study examined the SULTR gene family in soybean, analyzing the gene structure, conserved motifs, *cis*-acting elements, tissue expression patterns, and expression profiling under abiotic stress conditions.

The SULTRs of soybean [16], *Arabidopsis* [18], maize [15], sorghum [9], and apples [1] are generally divided into four subfamilies; 28 GmSULTR genes within each subgroup share similar motifs, gene structures, and *cis*-regulation elements, supporting the classification of subfamilies. Variations in the number of exons and introns within subfamilies may be attributed to gene functional diversity during evolution (Figure 2 and Figure 3). A collinearity analysis revealed that soybean has a higher homology with *Arabidopsis* and tomato (Figure 1). Currently, the research on sulfur transporters in *Arabidopsis* is more extensive, which also provided a reference for our research [31,32].

The expression patterns of GmSULTRs exhibit tissue specificity and responsiveness to various abiotic stresses and hormonal signals. In our investigation, group 3 obtained 13 sulfate transporter genes, which demonstrated differential expression across various soybean organs. *GmSULTR3;1a* exhibited a high transcript abundance in the roots, stems and leaves, and responded to low sulfur stress (Figure 4 and Figure 5F); similarly, *MdSultr3;1a* was especially expressed in the roots and was induced by low S [1], while *AtSULTR3;1* was preferentially expressed in the stems [21]. *GmSULTR3;4a* and *GmSULTR3;2b* were widely expressed in various organs and had high expression levels in the pods, and *GmSULTR3;3a and GmSULTR3;3c* had high expression levels in the pods and leaves. This was similar to the *Arabidopsis* homologous sulfate transporter genes At*SULTR3;2* and At*SULTR3;4,* which were expressed in multiple organs. The *AtSULTR3;3* gene was detected in mature seeds and leaves [21].

Additionally, *GmSULTR3;1a* was induced by drought, salt and alkali conditions in the roots (Figure 5A–C). Recent research has shown that various *SULTR3* genes play a role in responding to stress, including the *SULTR3;1* gene in the roots of Medicago truncatula and *Arabidopsis* under drought and salt stress [31]. In poplar, *SULTR3;3a* and *SULTR1;1* are utilized to reduce xylem offloading; meanwhile, ALMT3b is utilized to increase the load of parenchyma cells to the xylem, which is conducive to an improved sulfate content in xylem saps in order to cope with drought stress [6]. In our study, the *GmSULTR3;1a* gene increased the salt tolerance in yeast cells compared to the control cells (Figure 6), and the overexpression of *GmSULTR3;1a* in hairy roots conferred salt tolerance (Figure S2). Most *GmSULTRs* were upregulated by an ABA treatment, and their promoters contained ABREs, a key element for ABA crosstalk with stressors, similar to *AtSULTR3;1* expression [33,34]. Cao et al. proposed a role for *AtSULTR3;1* in helping plants to cope with environmental stresses by providing sulfate for the synthesis of cysteine, which serves as a sulfur donor during ABA biosynthesis [31]. Furthermore, MeJA-responsive elements were identified within the promoters of these genes, and the majority of *GmSULTRs* exhibited upregulation by exogenous MeJA (Figure 5E), similar to apple and cotton [14].

In response to the overexpression of *GmSULTR3;1a* in transgenic soybean hairy roots, the contents of methionine (Figure 7B), SO_4_^2−^ (Figure 7C), and GSH (Figure 7D) were significantly higher than those in the WT plant. *OsSULTR3*;*3* and *MhSULTR3*;*1a* play roles in SO_4_^2−^ homeostasis, metabolism, and partitioning processes [25]. This is consistent with a study reporting that *AtSultr1;1* in *Arabidopsis* and *LeST1-1* in tomato, which are mainly expressed in the roots and upregulated by S starvation, were primarily responsible for the root uptake of SO_4_^2−^ from the soil [35]. Moreover, studies have confirmed that the overexpression of *GmSULTR1;2b* genes could improve plant growth under low-S conditions by upregulating the genes involved in the S assimilation pathway and promoting the biosynthesis of essential amino acids and S-containing compounds [16]. In contrast, *ZmSultr3;1* was also specifically expressed in maize roots but was not affected by S deficiency [15]. These results suggest that *SULTR* exhibits species specificity. Meanwhile, the overexpression of *GmSULTR3;1a* could not facilitate an increase in biomass. *GmSULTR3;1a* participates in the absorption of SO_4_^2−^, promotes sulfur assimilation metabolism, and increases the content of methionine in plants when overexpressed.

## 4. Materials and Methods

### 4.1. Gene Structure, Conserved Motifs, and Promoter Analysis of GmSULTRs

Gene structure information was extracted from genome annotation files. Conserved motifs were analyzed using MEME 5.5.5 and *cis*-acting elements were identified using PlantCare and visualized with TBtools-II v2.096 [36].

### 4.2. Syntenic Analysis between Glycine and Other Species

A syntenic analysis between *Glycine* and four other species was conducted using the MCScanX program within Tbtools-II v2.096. Genome sequences and annotation files in the gff3 format were utilized as the input data to identify syntenic blocks for each species pair, with the default parameters employed.

### 4.3. Plant Materials and Treatments

Soybean tissues from “Dongnong50” were collected in 2023 at the Northeast Agricultural University. Samples of roots, stems, leaves, and pods at the V3 (the third trifoliolate leaf expansion), R2 (the blooming period), R7 (the initiation of maturity), and R8 (full maturity) growth stages were frozen in liquid nitrogen and stored at −80 °C for a gene expression analysis.

Seedlings at the V3 stage were subjected to abiotic stress and hormone treatments, including high salinity (150 mM NaCl), drought (15% PEG6000) and alkali conditions (75 mM NaHCO_3_), and ABA (5 µM) and MeJA (50 µM). Root and leaf samples were collected at various time points (0 h, 1 h, 3 h, 6 h, 12 h, and 24 h), immediately frozen in liquid nitrogen, and stored at −80 °C for a subsequent expression analysis.

For the sulfate deprivation treatment, the seedlings were treated in Hoagland’s nutrient solution (control, CK) or Hoagland’s nutrient solution lacking S supplemented with 0.1 mM of MgSO_4_ (low S treatment), and samples of the roots and leaves were collected at the V3 stage after the treatment.

### 4.4. Total RNA Extraction and qRT-PCR Analysis

RNA was extracted using Qiagen RNA Extraction Kits; 0.3 μg of RNA was used for cDNA synthesis using the TransScript^®^ One-Step SuperMix. qRT-PCR was performed on a LightCycler R 96 using the TransStart^®^ Top Green qPCR SuperMix. *GmTUA5* was used as the reference gene. After the initial denaturation at 95 °C for 30 s, the gene products were independently amplified using 40 cycles of 94 °C for 10 s and 60 °C for 30 s. The melt curve stage was 95 °C for 15 s, 60 °C for 1min, 95 °C for 1 s. All the primers used for the qRT-PCR are shown in Table S3. *GmTUA5* was used as the reference gene. Three biological and technical replications were assayed and the data were calculated using the $2^{-∆∆ct}$ method [37].

### 4.5. Stress Tolerance Analysis

The *GmSULTR3;1a* coding sequence was PCR amplified from Dongnong50 (DN50) leaf cDNA and inserted into a pYES2 vector before being introduced into the yeast strain INVSc1. The yeast cells were grown in SC-URA medium at 28 °C for 24 h and adjusted to different OD600 values to 1, 0.1, 0.01, and 0.001 [38]. The yeast cells were subjected to various stress tests, including salt stress (3 M NaCl), drought stress (2 M sorbitol), and alkali stress (0.8 M NaHCO_3_ on SC-URA medium at 28 °C). Transgenic yeast containing the empty vector pYES2 served as the control in the experiments, which were conducted in triplicate.

*GmSULTR3;1a*-overexpressing hairy root materials were treated with 150 mM of NaCl and phenotypic changes were observed after 20 h.

### 4.6. Preparation of Transgenic Soybean Hairy Roots

The *GmSULTR3;1a* coding sequences were inserted into pCAMBIA1300-35S-EGFP and introduced into *A. rhizogenes* K599. The soybean cultivar DN50 was used for the transformation under a 16 h light/8 h dark photoperiod at 25 °C in a humidity chamber, resulting in the growth of transformed hairy roots [39]. The overexpression of the transgene was verified in hairy roots by qRT-PCR analysis.

### 4.7. Determination of Quality Index of Transgenic Soybean Hairy Roots

The amino acid content, SO_4_^2−^ content, GSH (glutathione) content and biomass were measured. The amino acid content of the transgenic soybean hairy roots was analyzed using the national standard method of ion exchange chromatography (GBT15399-2018): https://openstd.samr.gov.cn/bzgk/gb/index (accessed on 31 May 2024). The concentration of SO_4_^2−^ was quantified following the methodology in [40]. The levels of GSH were assessed using the Solarbio company kit [41].

Transgenic soybean hairy roots were subjected to drying at 105 °C for 30 min, followed by further drying at 80 °C until a constant weight was achieved. Dry matter accumulation was determined through weighing. Additionally, the root length and number of lateral roots were quantified.

### 4.8. Statistical Analysis

The statistical analysis were conducted using the SPSS 23.0 software, with the experiments performed in triplicate and the results presented as the mean ± SD. Student’s *t*-test was used to generate the *p* values. The statistical significance was set at *p* < 0.05, and the graphs were created using GraphPad Prism 8.0.1.

## 5. Conclusions

A total of 28 GmSULTR genes were identified within the *Glycine* genome and classified into four distinct subfamilies. In this study, genes within the same subfamily exhibited similar gene structures and conserved motifs. Synteny analysis revealed an uneven distribution of *GmSULTR* genes across chromosomes, suggesting that fragment duplication significantly contributed to the expansion of the *GmSULTR* gene family. The expression of *GmSULTRs* was tissue-specific and induced by abiotic stresses and hormones; *GmSULTR3;1a* enhanced the uptake of SO_4_^2−^, thereby promoting sulfur assimilation metabolism. Additionally, it contributed to an increase in the amino acid content within the plants.

## Data Availability

Data are contained within the article and Supplementary Materials.

## Supplementary Materials

The following supporting information can be downloaded at: https://www.mdpi.com/article/10.3390/ijms25169080/s1.
