# Supplementary material for: Identification and Expression Analysis of Sulfate Transporter Genes Family and Function Analysis of GmSULTR3;1a from Soybean
Source: Int J Mol Sci. 2024 Aug 21;25(16):9080. doi: 10.3390/ijms25169080 (PMC11354235; doi:10.3390/ijms25169080)
Supplement: Supplementary file 1 [file ijms-25-09080-s001.zip › Figures S1 and S2.pdf]

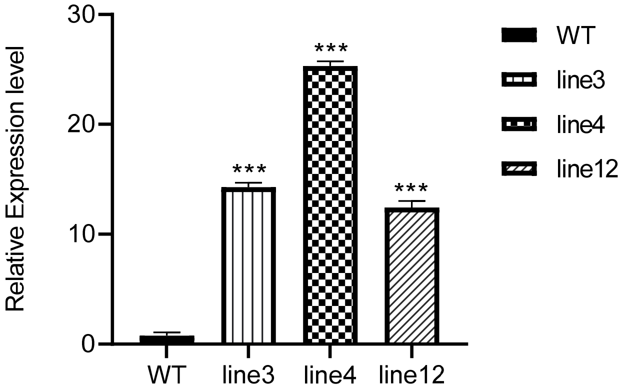

Figure S1. qRT-PCR analysis of *GmSULTR3;1a* transcript levels in transgenic hairy roots material (Line3, Line4, Line12). Asterisks indicate signifficant difference, ***P< 0.001) compared to control.

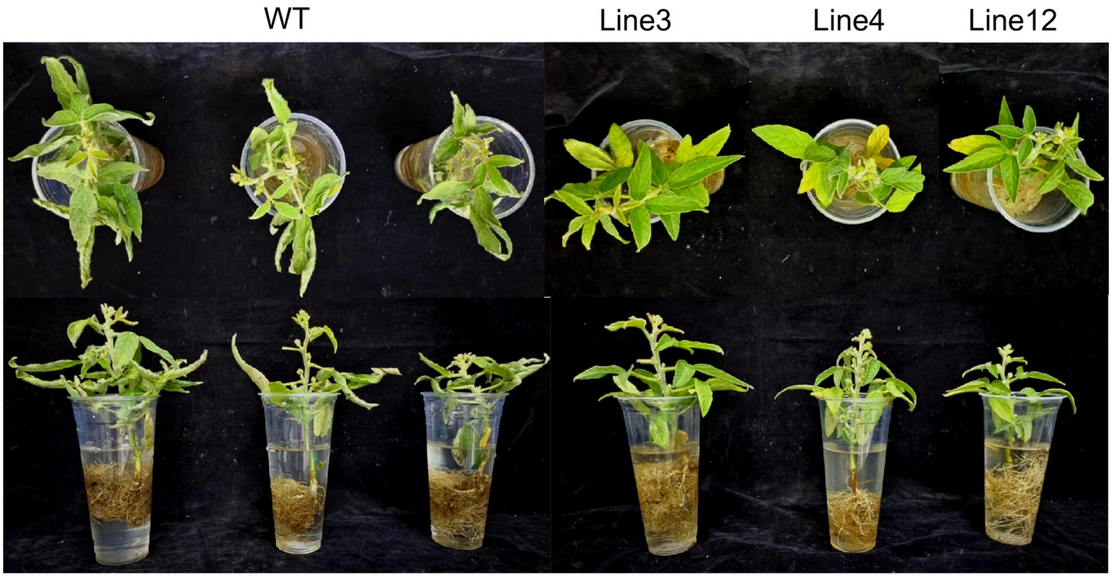

Figure S2. Phenotype of salt - treated (150 mM NaCl) of WT and *GmSULTR3;1a*-hairy root material (Line3, Line4, Line12) after 20 h.
